# Clinical evaluation of corneal changes after phacoemulsification in diabetic and non-diabetic cataract patients, a systematic review and meta-analysis

**DOI:** 10.1038/s41598-017-14656-7

**Published:** 2017-10-26

**Authors:** Yizhen Tang, Xinyi Chen, Xiaobo Zhang, Qiaomei Tang, Siyu Liu, Ke Yao

**Affiliations:** 1 Eye Center, Second Affiliated Hospital, School of Medicine, Zhejiang University, Hangzhou, Zhejiang, P.R. China; 2 Key Laboratory of Ophthalmology of Zhejiang Province, Hangzhou, P.R. China

## Abstract

Corneal endothelium morphological abnormalities result in fluid imbalance, stromal swelling, and loss of transparency, thus impairing visual function. Recently, growing number of studies have focused on diabetic corneal abnormalities after cataract surgery and its comparison with non-diabetic patients, the results remain conflicting. Thus, to evaluate the effect of phacoemulsification on the corneal properties in diabetic and non-diabetic patients, prospective studies were comprehensively searched through PubMed, EMBASE, and Cochrane databases updated to Jan 2017. A meta-analysis of the 13 identified studies was performed using weighted mean difference (WMD) and 95% confidence interval (CI). For the dynamic changes between preoperative and postoperative values, significant differences were identified between the two groups in endothelial cell density (ECD) and hexagon cells (HC%) at 1 day, 1 week, 1 month, and 3 months postoperatively, in central corneal thickness (CCT) at 1 month postoperatively, and in coefficient variation (CV) at 1 week and 1 month postoperatively. However, no significant differences were observed in CCT at 1 day, 1 week and 3 months postoperatively or in CV at 1 day and 3 months postoperatively. Diabetic corneas are more vulnerable to stress and trauma, resulting in greater morphological abnormalities and longer recovery time.

## Introduction

As of 2015, an estimated 415 million people had diabetes worldwide^1^, which is almost 1.5 times greater than in 2010 (285 million). The global prevalence of diabetes is growing much faster than earlier forecasts predicted (366 million by the year 2030)^2,3^. Obviously, diabetes mellitus (DM) is becoming more prevalent and threatening than was previously thought.

It has been acknowledged that DM leads to various complications such as nephropathy, neuropathy, cardiovascular issues, and several ocular complications like diabetic retinopathy, diabetic cataract, diabetic keratopathy, and diabetic optic nerve diseases^4^. Although the cornea may appear disease free in the diabetic, an awareness of the marked biochemical and ultrastructural abnormalities in the diabetic enables us to prevent more overt complications. The diabetic cornea suffers from endothelium cellular dysfunction and dysfunctional repair mechanisms including corneal edema, delayed wound healing, and so on^5,6^. Over the past decades, the pathology of the diabetic corneal endothelium dysfunction has become understood in more detail^7^. The state of hyperglycemia results in an increase in aldose reductase activity, the expression of metalloproteinase (MMP), and the formation of advanced glycation end products (AGEs). Evidence has shown that the inhibition of aldose reductase reduces dysmorphological changes in the corneal endothelium^8,9^. Enhanced MMPs can damage the basement membrane and limit cell migration, resulting in poor healing^10^. Moreover, the accumulation of AGEs can lead to an abnormality of cell adhesion^11^. The cornea is likely to be more vulnerable to stress and trauma in diabetic patients than in non-diabetics.

Phacoemulsification has become the predominant treatment procedure for cataract, the leading cause of blindness and visual impairment worldwide^12,13^. Although most cataract patients achieve decent recovery, unfortunately, in a complex disease environment, cataract surgery with phacoemulsification and lens implantation leads to larger endothelial cell loss in diabetic corneas^14–18^. Furthermore, the corneal endothelium can be adversely affected by surgery due to factors like lens nuclear sclerosis, effective phacoemulsification time (EPT), phacoemulsification energy, and IOL implantation^15,19–21^. These factors coupled with the effect of DM indicate a great risk of long-term endothelium cell dysfunction with decompensation and the development of bullous keratopathy^22^. Accelerated losses of corneal endothelial cells have been reported to continue even 10 years after surgery^23^.

To evaluate the corneal state, corneal thickness and endothelial cell morphology are the top two clinical concerns. Central corneal thickness (CCT), as an indicator of the physiological condition of the corneal endothelium, is generally used in diagnoses like keratoconus, Fuchs’ dystrophy, and glaucoma. Recognizing CCT is important because it can mask an accurate reading of intraocular pressure (IOP)^24^, causing doctors to unnecessarily treat for a condition that may not exist. Endothelial morphological changes in corneas, including endothelial density (ECD), coefficient of variation (CV), and percentage of hexagonal cells (HC%), can alter the cornea’s ability to function. Abnormal corneal endothelial cell morphology coupled with increased CCT^25^ is another marker of endothelial cell dysfunction, which results in fluid imbalance, stromal swelling, and loss of transparency, thus impairing visual function.

Recently, a growing number of studies have focused on the importance of diabetic corneal abnormalities, which were commonly found in patients after cataract surgery due to factors including diabetic state and surgical procedures^26^. However, there is still conflict concerning the differences in corneal properties between diabetic and non-diabetic patients after phacoemulsification. According to our knowledge, there has been no comprehensive review or meta-analysis regarding corneal changes after phacoemulsification for diabetic and non-diabetic groups so far. Under the circumstances, this article is set to evaluate the effect of phacoemulsification on ECD, HC%, CV, and CCT in diabetic patients and non-diabetic patients.

## Results

### Literature selection

**Figure 1:**
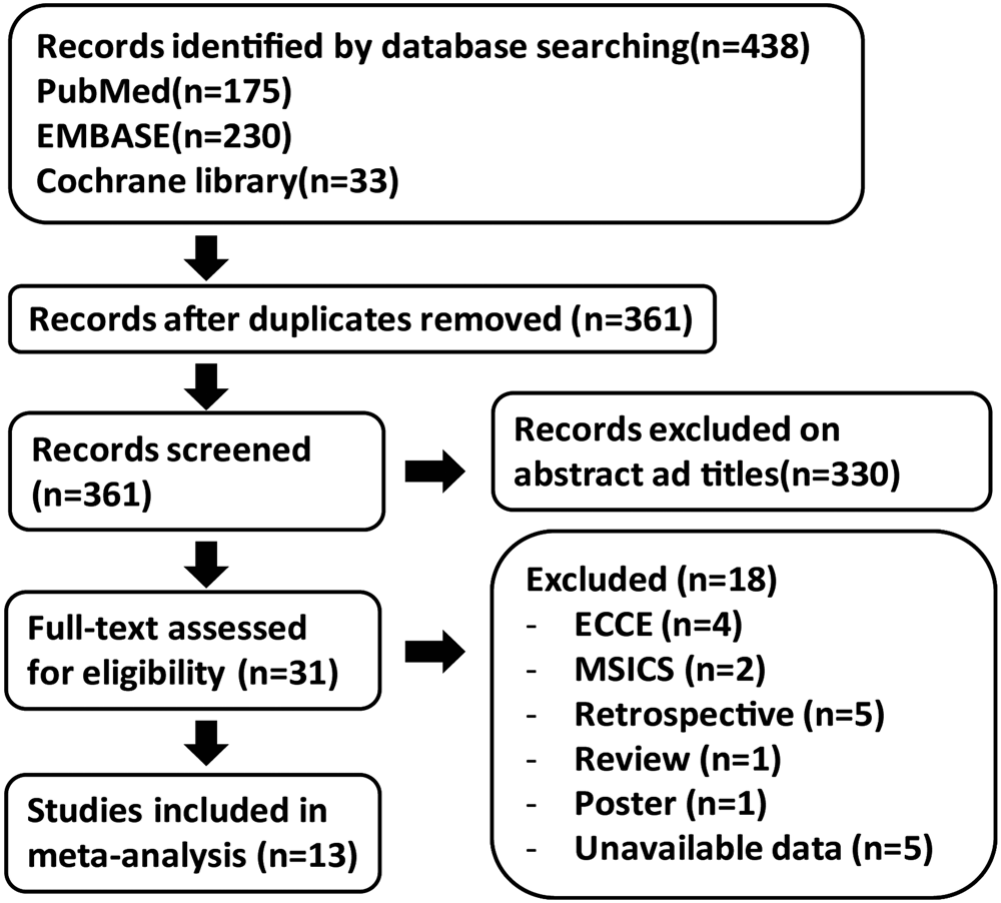
Workflow chart of the literature selection process.

The workflow chart in Fig. 1 shows the literature selection process. After duplication removal, a total of 361 studies were retrieved from the databases. 330 studies were excluded by scanning titles and abstracts. Furthermore, 18 studies were excluded after full-text reading: four on extracapsular cataract extraction (ECCE), two on manual small incision cataract surgery (MSICS) which is different from phacoemulsification from either the incision size or the surgical method, five retrospective studies, five with unavailable data (e.g. no postoperative data, no cohort, or an unmatched comparison group), one that was a poster, and one that was a review. Finally, 13 studies^17,19,27–37^ meeting all of the predefined criteria were identified. The characteristics of the included studies are shown in Table 1. The details of N OS scale are shown in Table S6.

**Table 1:** Characteristics of included studies. NA = not available, DM = diabetes mellitus, NOS = The Newcastle–Ottawa scale.

Study ID	Country/province	Study design	Follow up duration	No. of eyes Gender (M/F)		Age (year/SD, range)		Diabetes condition	Quality control (NOS)
				Normal	DM	Normal	DM		
Li^19^	China Hainan	Prospective controlled study	3 months	106/84	101/85	65.1 (12.3)	64.6 (12.5)	NA	7
Misra^27^	New Zealand	Prospective cohort study	6 months	23	28	74.4 (7.4)	71.2 (7.6)	Duration (11.54 years) HbA1c% (DM:nDM 7.5:5.7, P < 0.001)	8
Zhu^28^	China Beijing	Prospective controlled study	1 month	16/17	15/20	60–80	60–80	Blood glucose < 8.3 mmol/L, diabetic course > 5a	8
Yan^29^	China Guizhou	Prospective controlled study	3 months	30/32	51/46	69.6 (55–85)	65.6 (52–82)	Diabetic course > 10a, Diabetic course < 10a	8
Su^30^	China Liaoning	Prospective controlled study	1 month	31	39	50–80	50–80	Blood glucose < 8.3 mmol/L, diabetic course < 15a	8
Liu^31^	China Hebei	Prospective controlled study	1 week	16/14	17/13	64.7 (3.6)	69.6 (5.4)	NA	8
Zhao^32^	China Shandong	Prospective controlled study	3 months	24/26	26/24	64.8 (55–83)	63.2 (52–80)	NA	8
Wang^33^	China inner Mongolia	Prospective controlled study	6 months	36/24	24/16	69.2 (8.2)	65.3 (11)	NA	7
Yang^34^	China Guangzhou	Prospective controlled study	3 months	33/32	34/41	68.1 (50–84)	67.8 (51–83)	Blood glucose < 6. 6–10 mmol/L Duration (1–17 years)	8
Hugod^35^	Denmark	Prospective controlled study	3 months	30	30	75.4 (9.3)	75.6 (8.6)	HbA1c%(7.08 (1.43))	8
Wu^36^	China Shaanxi	Prospective controlled study	3 months	31	28	50–80	50–80	Diabetic course < 12a	7
Wu^37^	China Hainan	Prospective controlled study	3 months	33/31	26/24	65.6 (52–82)	66.8 (50–84)	NA	8
Morikubo^17^	Japan	Prospective controlled study	1 month	93	93	68.8 (8.9)	68.6 (8.8)	NA	8

### Meta-analysis of the outcomes

A total of 13 prospective studies including 1923 eyes (941 in the non-diabetic group and 982 in the diabetic group) were identified. The surgical parameters (e.g. phaco-time and phaco-energy) and lens nucleus hardness were reported with no significant differences between the DM and non-DM groups, as shown in Tables S1–S4.

### Endothelial cell density

**Figure 2:**
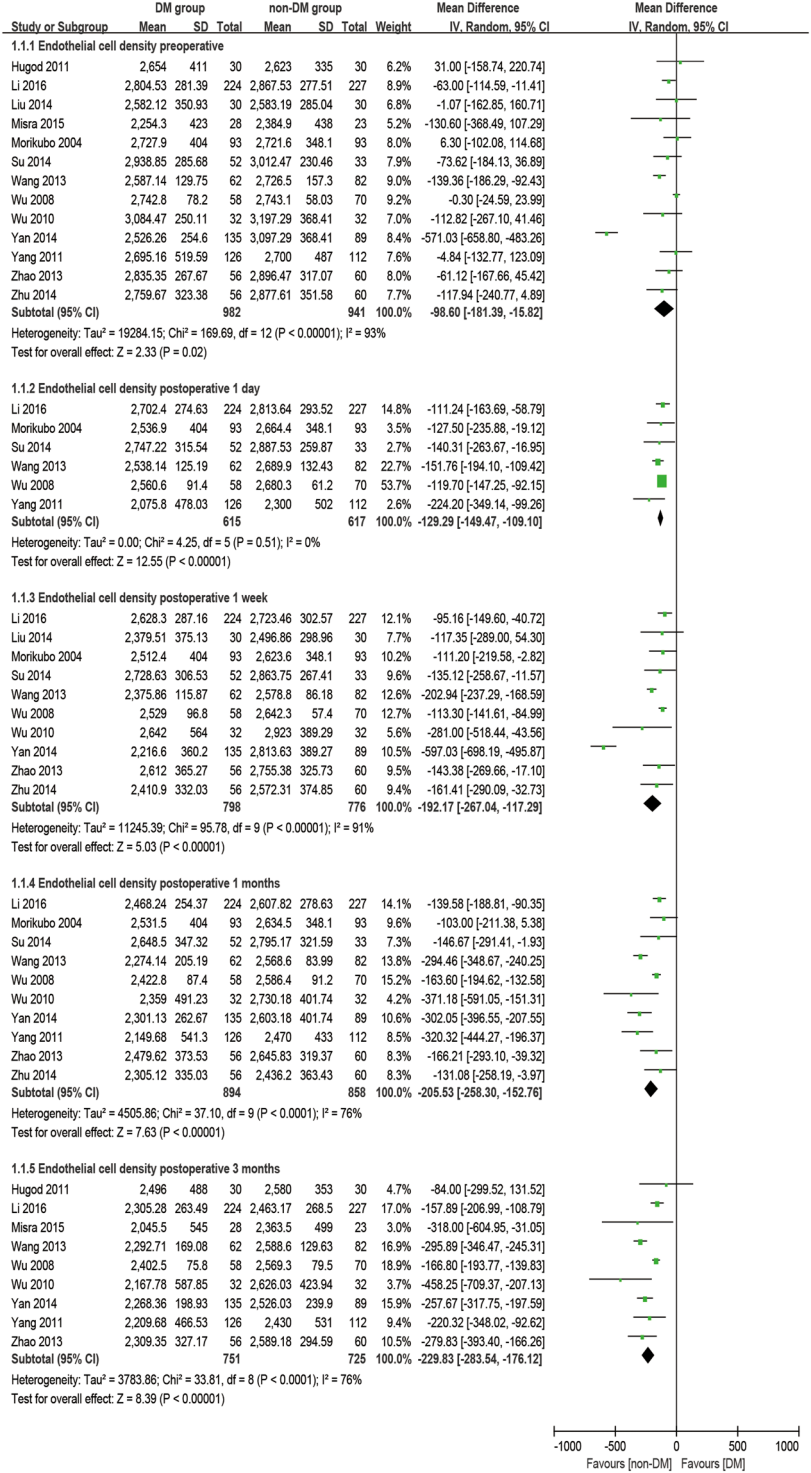
Forest plot comparison of the corneal endothelial cell density (ECD) after phacoemulsification in diabetic and non-diabetic patients.

**Figure 3:**
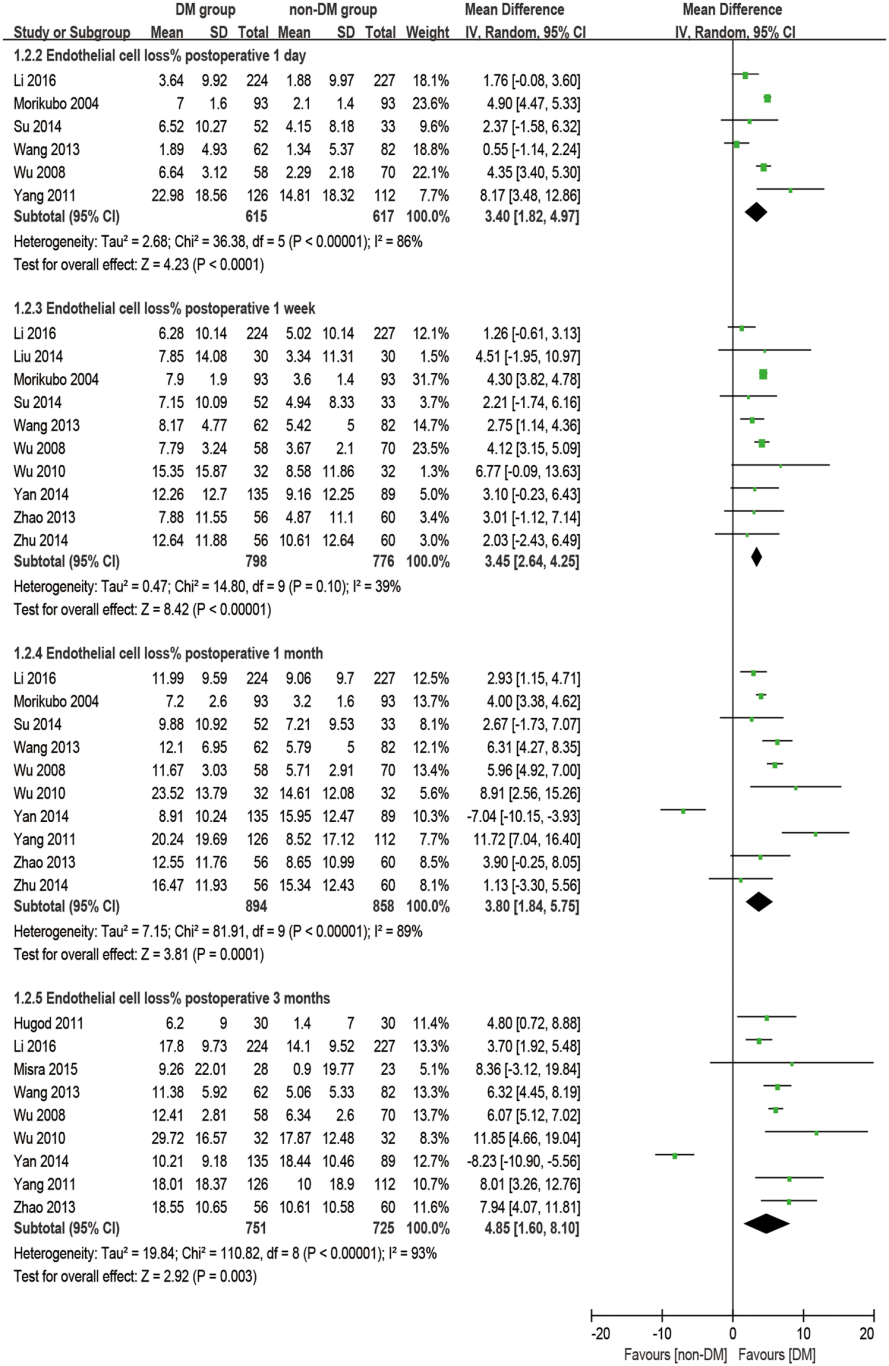
Forest plot comparison of the corneal endothelial cell loss in percentage (ECL%) after phacoemulsification in diabetic and non-diabetic patients.

There were thirteen studies reporting the outcome of ECD. The analysis was made at 1 day, 1 week, 1 month, and 3 months postoperatively. It was found that diabetic patients have a significantly lower ECD at preoperative and all postoperative time points than the non-diabetic group (baseline: WMD = −98.60, 95% CI: −181.39 to −15.82, P = 0.02; 1 day postoperative: WMD = −129.29, 95% CI: −149.47 to −109.10, P < 0.001; 1 week postoperative: WMD = −192.17, 95% CI: −267.04 to −117.29, P < 0.001; 1 month postoperative: WMD = −205.53, 95% CI: −258.30 to −152.76, P < 0.001; 3 months postoperative: WMD = −229.83, 95% CI: −283.54 to −176.12, P < 0.001; Fig. 2). Furthermore, the percentage of the loss of ECD (ECL%, difference between preoperative and postoperative), which was calculated from equations (1–5), was also evaluated to see the effect caused by phacoemulsification. There are significant differences in ECL% at all postoperative times for the DM group compared to the non-DM group (1 day postoperative: WMD = 3.40, 95% CI: 1.82 to 4.97, P < 0.001; 1 week postoperative: WMD = 3.45, 95% CI: 2.64 to 4.25, P < 0.001; 1 month postoperative: WMD = 3.80, 95% CI: 1.84 to 5.75, P < 0.001; 3 months postoperative: WMD = 4.85, 95% CI: 1.60 to 8.10, P = 0.003; Fig. 3).

### Coefficient of variation

**Figure 4:**
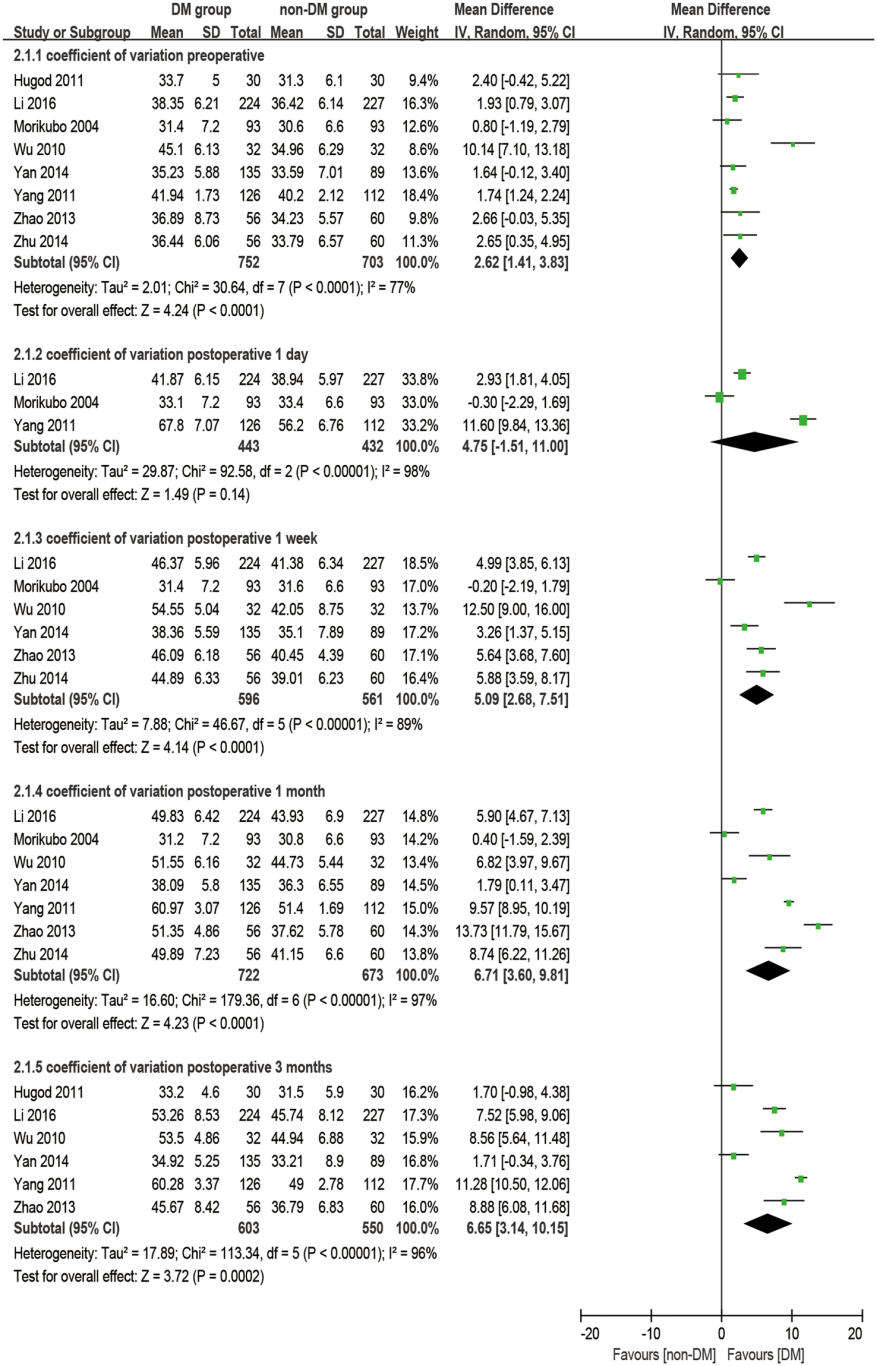
Forest plot comparison of the coefficient of variation (CV) of corneal endothelial cells after phacoemulsification in diabetic and non-diabetic patients.

**Figure 5:**
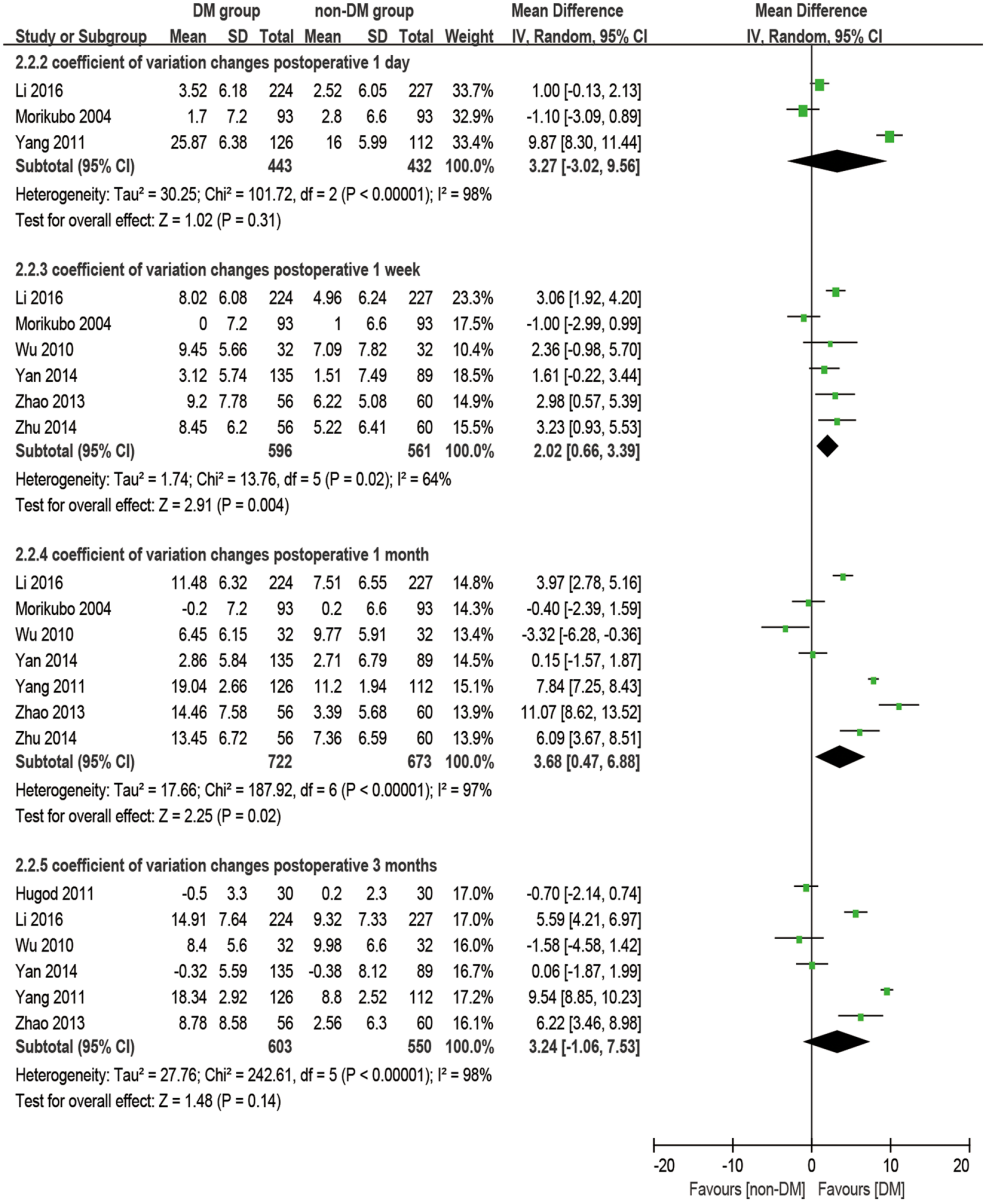
Forest plot comparison of the change of coefficient of variation (dCV) of corneal endothelial cells after phacoemulsification in diabetic and non-diabetic patients.

There were eight studies reporting the outcome of coefficient of variation. The analysis was made at 1 day, 1 week, 1 month, and 3 months postoperatively. It was observed that diabetic patients had a significantly higher CV at preoperative and all postoperative time points than the non-DM group (baseline: WMD = 2.62, 95% CI: 1.41 to 3.83, P < 0.001; 1 week postoperative: WMD = 5.09, 95% CI: 2.68 to 7.51, P < 0.001; 1 month postoperative: WMD = 6.71, 95% CI: 3.60 to 9.81, P < 0.001; 3 months postoperative: WMD = 6.65, 95% CI: 3.14 to 10.15, P < 0.001), except at 1 day postoperatively (WMD = 4.75, 95% CI: −1.51 to 11.00, P = 0.14, Fig. 4). The increase of CV (dCV, difference between preoperative and postoperative) appears significantly larger in the DM group compared to the non-DM group at 1 week postoperatively (WMD = 2.02, 95% CI: 0.66 to 339, P = 0.004) and at 1 month postoperatively (WMD = 3.68, 95% CI: 0.47 to 6.88, P = 0.02), while no significant differences were found at 1 day postoperatively (WMD = 3.27, 95% CI: −3.02 to 9.56, P = 0.31) and 3 months postoperatively (WMD = 3.24, 95% CI: −1.06 to 7.53, P = 0.14, Fig. 5).

### Hexagonal cell percentage

**Figure 6:**
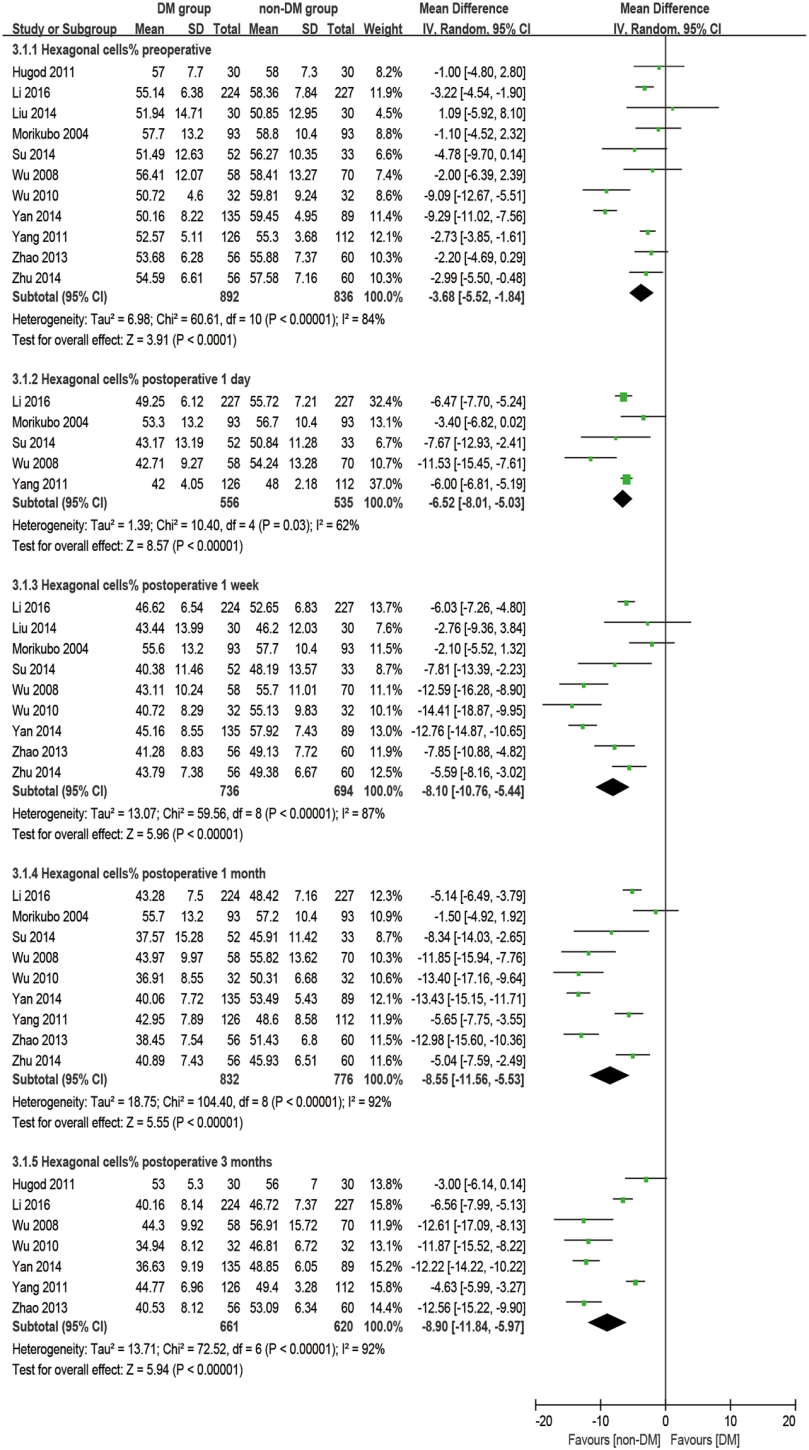
Forest plot comparison of the percentage of hexagonal corneal endothelial cells (HC%) after phacoemulsification in diabetic and non-diabetic patients.

**Figure 7:**
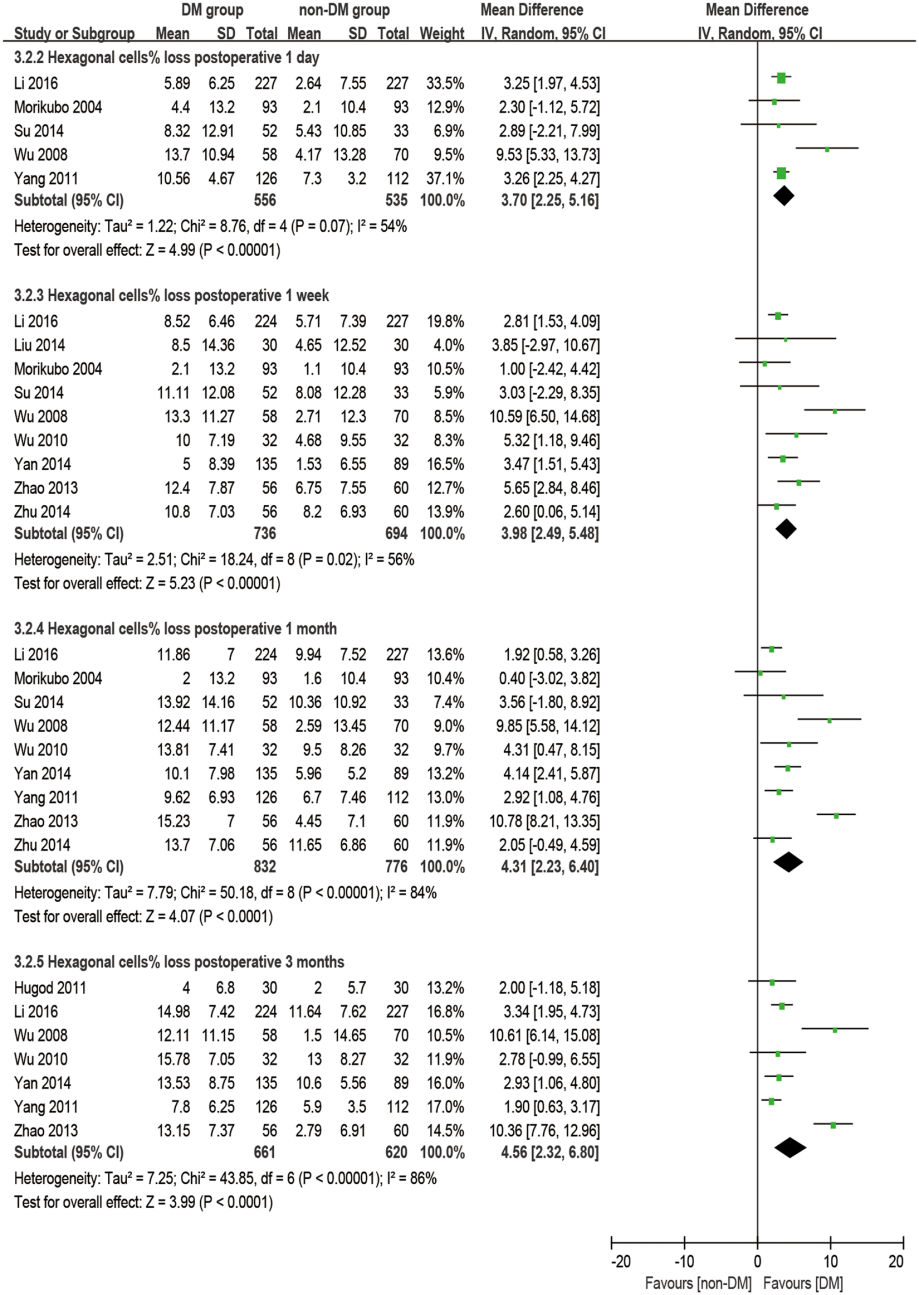
Forest plot comparison of the loss of percentage of hexagonal corneal endothelial cells (dHC%) after phacoemulsification in diabetic and non-diabetic patients.

There were 11 studies reporting the outcome of hexagonal cells. The analysis was made at 1 day, 1 week, 1 month, and 3 months postoperatively. It was found that diabetic patients have a significantly smaller HC% at preoperative and all postoperative time points (all: P < 0.001, Fig. 6) and a significantly larger HC% loss (difference of preoperative and postoperative) at all postoperative times (all: P < 0.001, Fig. 7) compared to the non-DM group.

### Central corneal thickness

**Figure 8:**
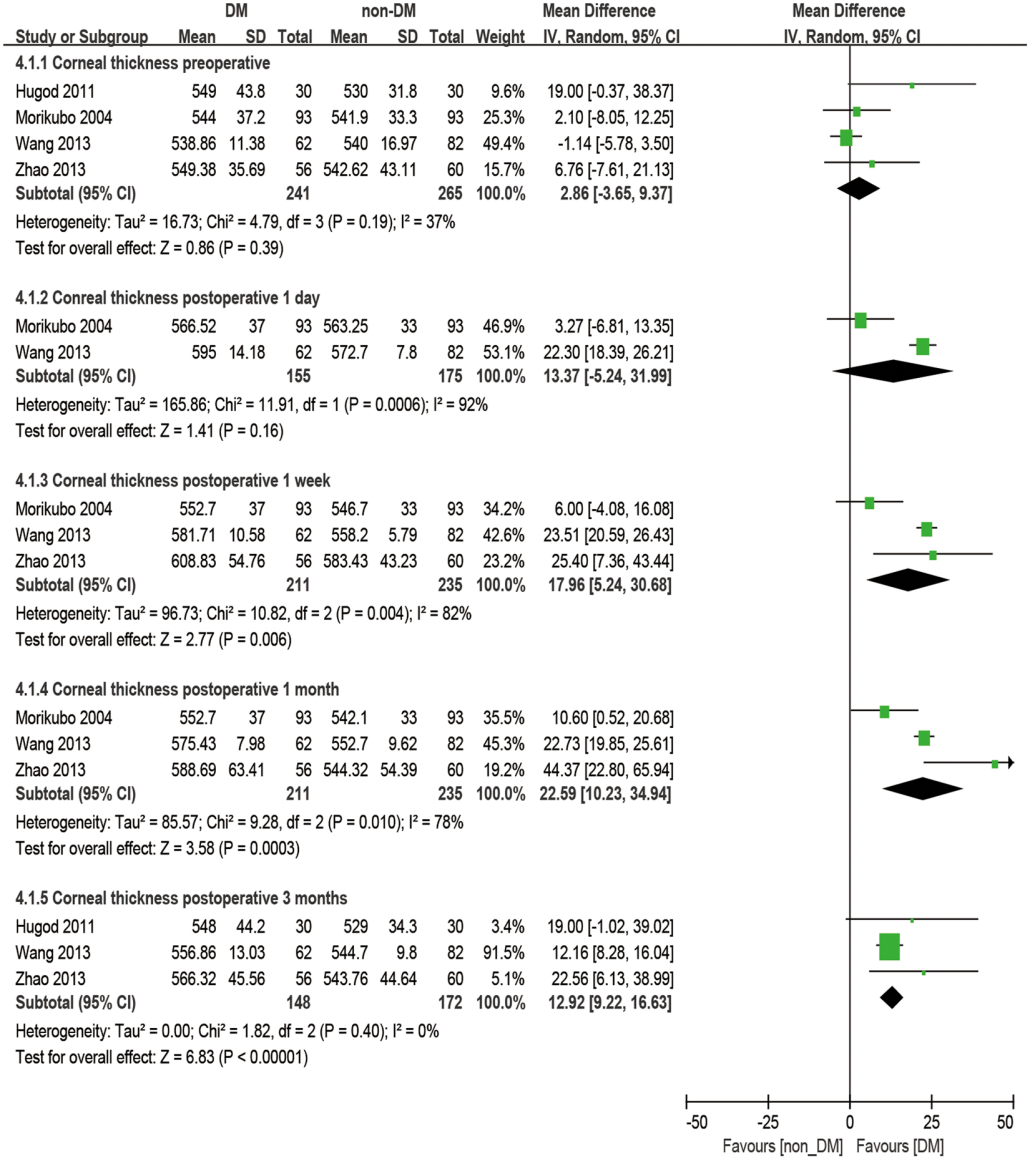
Forest plot comparison of the central corneal thickness (CCT) after phacoemulsification in diabetic and non-diabetic patients.

**Figure 9:**
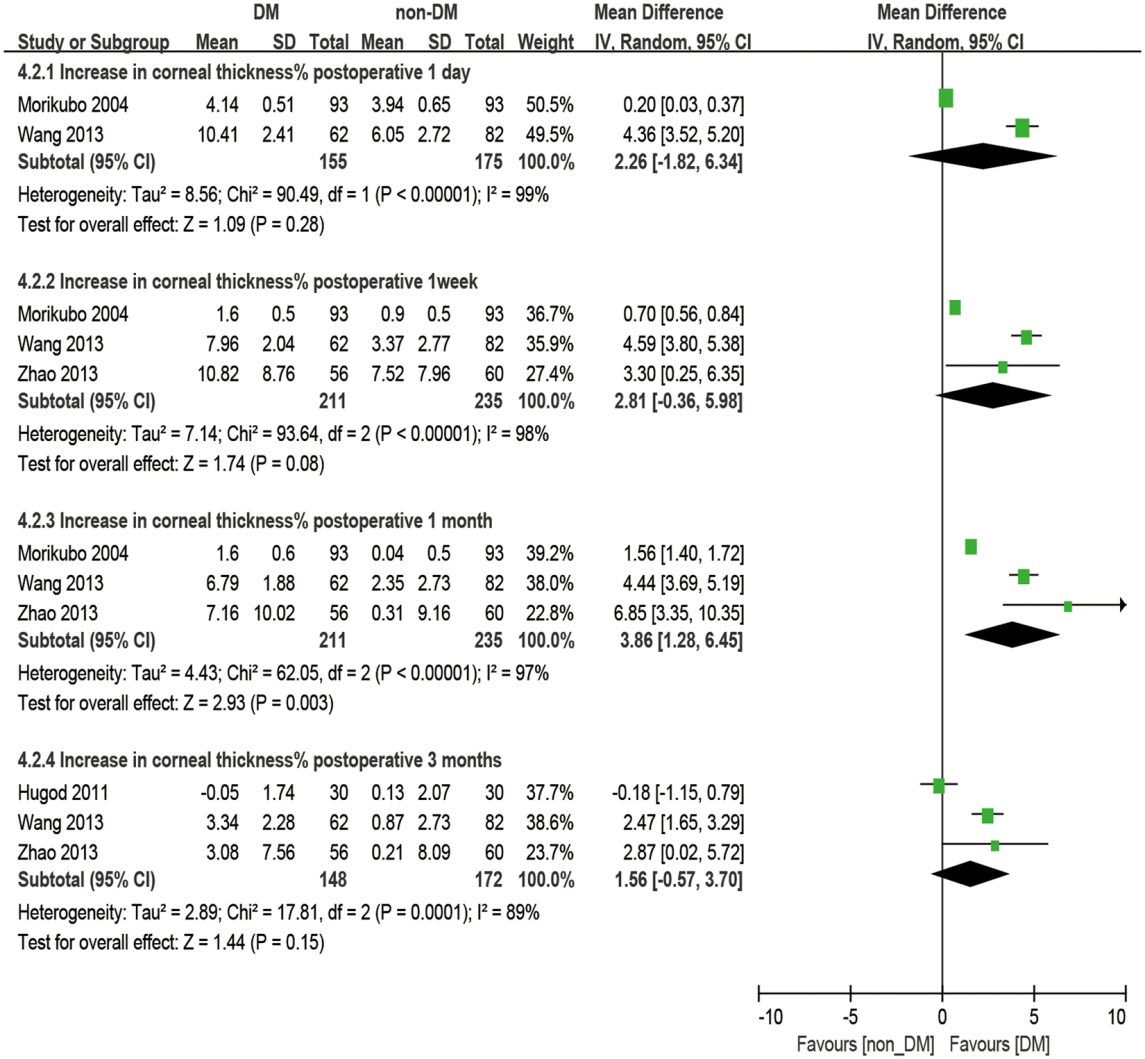
Forest plot comparison of the increased central corneal thickness percentage (dCCT%) after phacoemulsification in diabetic and non-diabetic patients.

There were four studies reporting the outcome of CCT. The analysis was made at 1 day, 1 week, 1 month, and 3 months postoperatively. No significant difference was observed in CCT between diabetic patients and non-diabetic patients. (baseline: WMD = 2.86, 95% CI: −3.65 to 9.37, P = 0.39, 1 day postoperative: WMD = 13.37, 95% CI: −5.24 to 31.99, P = 0.16). After phacoemulsification, the CCT of the DM group was significantly higher than that of the non-DM group at all postoperative time points (1 week postoperatively: WMD = 17.96, 95% CI: 5.24 to 30.68, P = 0.006; 1 month postoperatively: WMD = 22.59, 95% CI: 10.23 to 34.94, P < 0.001; 3 months postoperatively: WMD = 12.92, 95% CI: 9.22 to 16.63, P < 0.001; Fig. 8). Moreover, the percent increase of CCT (dCCT%, difference between preoperative and postoperative percentages) showed no significant difference between the DM group and the non-DM group at 1 day postoperatively (WMD = 2.26, 95% CI −1.82 to 6.43, P = 0.28), 1 week postoperatively (WMD = 2.81, 95% CI: −0.36 to 5.98, P = 0.08) and 3 months postoperatively (WMD = 1.56, 95% CI: −0.57 to 3.70, P = 0.15), but a significantly larger dCCT% was found in diabetic patients at 1 month postoperatively compared to the non-diabetic ones (WMD = 3.86, 95% CI: 1.28 to 6.45, P = 0.003, Fig. 9).

### Sensitivity analysis and publication bias

Since some of the results show heterogeneity (${I}^{2} > 50$), a random-effect meta-regression model was chosen to analyze this condition. No publication bias was found through Begg’s and Egger’s test as shown in Table S5. Outcomes included in only 3 studies are too small to do a sensitivity analysis so as to exclude trials at a high risk of bias. Thus, one-study-removed analyses were conducted for all remaining outcomes. The sensitivity analysis revealed that there are two pooled outcomes lacking stability: the outcome of ECD at the baseline and the outcome of dCV at 1 month postoperatively. Yan and Chen (2014)^29^, whose patients had a long duration of DM, is the source of the statistical heterogeneity in the meta-analysis of the preoperative ECD result as shown in Table 2. When this outlier study is removed, the outcome is stable, indicating that the outcome is still rational and reliable. The heterogeneity of the second sensitive outcome may be due to several design differences among the studies that affect the response degree and recovery duration, such as duration of diabetes and blood glucose control. No significant publication bias was demonstrated in the funnel plot.

**Table 2:** One-study-removed analysis for the outcomes of ECD preoperatively and dCV at 1 month postoperatively.

Study	P	95% CI	*I* ^2^
Endothelial cell density preoperatively			
Hugod^35^	P = 0.01	[−193.35, −21.02]	93%
Li^19^	P = 0.04	[−197.90, −4.74]	94%
Liu^31^	P = 0.02	[−192.48, −18.93]	93%
Misra^27^	P = 0.03	[−182.39, −11.18]	94%
Morikubo^17^	P = 0.02	[−195.75, −19.36]	93%
Su^30^	P = 0.03	[−189.02, −12.17]	94%
Wang^33^	P = 0.05	[−189.35, 1.16]	93%
Wu^37^	P = 0.03	[−202.90, −12.37]	91%
Wu^36^	P = 0.03	[−184.39, −10.50]	93%
Yan^29^	P = 0.01	[−100.10, −12.55]	67%
Yang^34^	P = 0.02	[−193.79, −18.58]	93%
Zhao^32^	P = 0.02	[−190.32, −13.07]	94%
Zhu^28^	P = 0.03	[−184.61, −9.14]	93%
Changes of coefficient of variation at 1 month postoperatively			
Li^19^	P = 0.09	[−0.50, 7.73]	97%
Morikubo^17^	P = 0.009	[1.09, 7.64]	97%
Wu^36^	P = 0.003	[1.59, 7.93]	97%
Yan^29^	P = 0.01	[0.99, 7.58]	96%
Yang^34^	P = 0.09	[−0.45, 6.33]	94%
Zhan^32^	P = 0.16	[−0.97, 5.94]	97%
Zhu^28^	P = 0.08	[−0.34, 6.90]	97%

## Discussion

The results of the present meta-analysis provided robust evidence that the effect of phacoemulsification on corneal changes in diabetics is greater than for non-diabetics. Significant differences have been observed between the diabetic and non-diabetic groups in terms of ECD, HC%, CV, and CCT preoperatively and 1 day, 1 week, 1 month, and 3 months postoperatively, except CV at 1 day postoperatively and CCT preoperatively. For the changes between the preoperative and postoperative state, significant differences were identified in ECL% and HC% loss at 1 day, 1 week, 1 month, and 3 months postoperatively, in dCCT% at 1 month postoperatively, and in dCV at 1 week and 1 month postoperatively between the two groups. Nevertheless, no significant differences were observed in dCV and dCCT% at 1 week and 3 months postoperatively.

### Corneal endothelial cell morphology

It was once considered controversial that diabetes could affect corneal endothelium morphology preoperatively. Inoue *et al*.^38^ investigated 1394 patients before cataract surgery and their multiple regression analysis revealed that age instead of DM was the only variable relevant to ECD, CV, and HC%. However, Lee *et al*.^39^ reported that corneal endothelium morphology was significantly different between DM and non-DM patients, and CV is significantly correlated with diabetes duration. Taking multiple studies into account, the results of this review offer the judgment that DM patients have lower ECD and HC%, but higher CV, than non-DM patients (CV: P < 0.001; ECD: P = 0.02; HC%: P < 0.001; Figs 2, 4 and 6) before phacoemulsification.

The fragility of the corneal endothelium in the eyes of diabetic patients might be explained by several mechanisms. With an enhanced polyol pathway and the accumulated sugar alcohol in cells converted by excessive glucose, the osmotic pressure goes up, causing the fragility of diabetic corneal endothelial cells. Morphological abnormalities in the corneal endothelium have been reported^8,40^ to improve after administration of an aldose reductase inhibitor in the polyol pathway, which supports its involvement in the corneal endothelial abnormalities of patients with DM. Furthermore, the enhanced accumulation of AGEs in diabetic corneas provides strong evidence^22^ that nuclear oxidative DNA damage caused by the accumulation of AGEs is responsible for the apoptotic damage of corneal endothelial cells in diabetic patients, which also results in decreased ECD. Diabetes also reduces the activity of Na^+^/K^+^-ATPase of the corneal endothelium^41^, which plays a key role in the maintenance of its structure. This causes morphological and functional changes, including increased CV and decreased HC% in diabetic corneas. Since a regular hexagonal pattern of the corneal endothelium provides the most stable covering plane, deviation from this pattern leads to a less stable monolayer. In the diabetic endothelium, there is a greater surface tension on the monolayer caused by the loss of the regular hexagonal pattern and the increasingly irregular shapes of the corneal endothelium, which makes the corneas of diabetic patients more fragile^26,42^. According to quantitative morphometric analysis, the corneas of patients with DM may be at risk in intraocular surgical procedures.

### Endothelial cell density

Clinical observations have indicated that the corneal endothelium is capable of compensation to prevent complicated diseases of the cornea, such as bullose keratopathy^22^, unless the cell density reaches a very low threshold of 400–500 cells/mm^2^, at which point the cornea cannot maintain its normal physiological function^43^. Typically, it is widely accepted that 1000 cells/mm^2^ is the minimum preoperative value to prevent corneal decompensation after surgery. The diabetic cornea, which is more fragile and vulnerable to trauma, possesses a weaker compensatory capacity. The study by Furuse *et al*.^44^ reportedfwhich point the cornea cannot

that there is no significant difference in the ECL% and CV values beween the two groups postoperatively. However, a recent study by Dhasmana *et al*.^45^ showed a severe increase in ECL% in the DM group compared to the control after cataract surgery. The results of our meta-analysis showed that DM patients have a significantly greater ECL% than non-DM patients from the first day to 3 months postoperatively (P < 0.01, Fig. 3), confirming that diabetic patients are more susceptible to corneal endothelial damage after phacoemulsification.

### Coefficient of variation and hexagonal cell percentage

It’s common practice that ECD is used to evaluate the state of corneas after phacoemulsification, but it cannot reflect the dynamic of the healing process for trauma. The change in morphology has a closer relationship with the dynamic of the corneal recovery process. The loss of endothelial cells, as an immediate response to surgery, leads to some defeats. Unlike the corneal epithelium, the cells of the endothelium do not regenerate. Instead, the remaining cells enlarge and stretch to cover the posterior corneal surface in order to fill the space. Ideally, the earliest phenomena should be an increase in cell size coupled with an enlargement in CV and a decrease in HC%. After a period of rearrangement, the defects would diminish, and CV and HC% would return to preoperative values as well^46^. Gradually, the cells return to stability to maintain the physiological function of corneas. Although many studies^28–30,32,34–37,45,47^ have mentioned that a significant difference in CV and HC% were found between DM and non-DM groups or between postoperative and preoperative periods, quite a few studies discuss the comparison of the dynamic change of CV and HC% between the two groups. Our meta-results showed that dCV follows a pattern of maximum increase between 1 day and 1 week postoperatively and then slowly reduces for at least 3 months. On the first day after phacoemulsification, both groups have an enlarged inhomogeneity in cell size, which gives two large dCV values without a significant difference (P = 0.31). The ascending process lasts for 1 day^17,34^ to 1 month^32^ before cell shape starts to compensate to be uniform. Significant differences between the two groups started at 1 week postoperatively (P = 0.004), peaked at 1 month postoperatively (P = 0.02, Fig. 7), and then vanished at 3 months postoperatively with the recovery of the diabetic patient (P = 0.14, Fig. 5). However, a sensitivity analysis showed an unstable outcome at 1 month postoperatively. It can be inferred that this is because the measurements were done at some critical point when the significant difference started to appear or disappear. What’s more, different durations of DM^29^ and blood glucose control^34,48^ might affect the result.

This kind of recovery was not uniform for all corneal morphological properties. For HC% loss, there was always a significant difference between the DM and non-DM groups postoperatively (P < 0.001, Fig. 7). Once the endothelial cells lose their hexagonal structure, the stretching and rearrangement process will not easily give a second chance for the cells to stabilize into hexagons again. The time of the HC% recovery in diabetic corneas should be longer than 3 months.

### Central corneal thickness

For the preoperative state, Kotecha *et al*.^49^ found no significant difference in CCT between the DM and non-DM groups. However, Lee *et al*.^18,39^ reported that CCT, which is strongly correlated with DM duration, is slightly greater in diabetic patients than in non-diabetic patients. The analysis revealed a slightly greater CCT in patients with DM, but no significant difference preoperatively (P = 0.39, Fig. 8).

The effect of DM on CCT is still ambiguous. There are several possible explanations, such as the inhibition of endothelial pumping, growing stromal swelling pressure, and increasing endothelial permeability caused by diabetic metabolism^50–53^. Briefly, the normal corneal endothelium plays a key role in keeping the cornea moist and transparent as well as maintaining integrity to prevent stromal swelling. Tight apical junctions on the endothelial cells function as physical barriers. The movement of water outward from the corneal stroma into the anterior chamber is increased due to ion pumps in the endothelial cells. Thus, corneal edema can be caused by a breakdown of either the anatomical barrier or the pump function of the corneal endothelial cells, representing an increase in CCT. This effect depends on the pathology insults of DM^18^ and the severity of the physical trauma.

After phacoemulsification, the increase in CCT was maximum at 1 day and 1 week postoperatively and then gradually decreased for at least 3 months^17,26,32,33,45,47^. Altintas *et al*.^26^ demonstrated that corneal thicknesses were greater in both diabetic and non-diabetic patients 1 week postoperatively than in later follow up, while there were no differences in corneal thickness according to phaco-time or diabetic status. Nevertheless, most studies^17,32,33,45,47^ mentioned a delayed recovery of postoperative corneal edema in diabetics compared to normal controls. This analysis observed significant differences between the two groups at all postoperative times from 1 day to 3 months. For the postoperative changes, the dCCT% followed the same trend as dCV. Severe postoperative responses and long recovery times in the diabetic group are shown by the analysis in Fig. 3. There was a significant difference found at 1 month postoperatively. It can be inferred that this is because, at an early time after phacoemulsification, both DM and non-DM patients have severe responses and sharp increases due to the breakdown of corneal endothelial function caused by the surgical procedure, thus making the difference between the two groups too small to be distinguished (P = 0.28, P = 0.08, Fig. 9). Furthermore, studies have proven that hyperglycemia enhance the expression of MMPs^54^, the production and activity of which is likely to damage the basement membrane, including type IV collagen, and limit epithelial cell migration, resulting in poor epithelial healing^55^. Gradually, the corneas of non-diabetic patients start to heal more quickly, showing a smaller CCT compared to the DM group, especially at 1 month postoperatively (P = 0.003, Fig. 9). Finally, differences due to the effect of surgery vanish 3 months postoperatively (P = 0.15, Fig. 9), which means DM patients can take almost 3 months to recover.

### Visual acuity

Besides all of the changes in the cornea, visual rehabilitation is still the top concern for patients undergoing phacoemulsification. Best corrected visual acuity (BCVA) is one of the best parameters for evaluating the quality and efficiency of a surgical technique. Although CCT values were significantly different between the two groups after phacoemulsification, there was no difference in visual acuity in the long-term comparison as reported^27,33,45^. However, the BCVA of the non-diabetic group was better at 1 week postoperatively^33^, indicating that the diabetic achieve worse vision recovery, which is consistent with the CCT results. Eventually, patients in both groups had better postoperative visual acuity at the end of the follow-up period, which indicates that phacoemulsification should be considered as a safe procedure for cataract extraction in the diabetic.

As a consequence, greater efforts and concentration should be made by the surgeon to minimize surgical trauma, especially for the diabetic. To achieve that, phaco-power near the cornea should be avoided. A viscoelastic agent could be generously used to cushion the endothelium as well. What’s more, close postoperative observation and intervention is suggested in patients with transient corneal edema and decompensation, as it can be a predicted factor for the development of pseudophakic cystoid macular edema^56^. Since the duration of DM and blood glucose have proven to be associated with the severity of corneal damage caused by phacoemulsification^29,48^, diabetic patients are recommended to choose the proper timing when good glycemic and HbA1c control is achieved for cataract surgery^35,48^, thereby preventing further complications and minimizing visual loss. Femtosecond laser-assisted cataract surgery is currently reported to be safer and more effective in reducing endothelial cell loss and postoperative central corneal thickening and achieving better visual and refractive outcomes compared to conventional phacoemulsification surgery. Therefore, it might be a potentially better choice for the diabetic. Further studies are needed to validate this hypothesis.

To the best of our knowledge, this is the first meta-analysis and review of corneal changes after phacoemulsification in diabetic and non-diabetic patients. Not only the postoperative state but also the changes between preoperative and postoperative states, which have an advantage over the former, are evaluated. In addition, the dynamic healing process and the changes of these parameters are carefully demonstrated in this study with at least three studies in each analysis to give a rational analysis result. We offer a systematic evaluation as well as possible mechanisms and treatment to the clinic.

Inevitably, this meta-analysis has several limitations. First, the limitations came from the clinical trial itself. It was not possible to have a randomized control trial (RCT) as diabetic patients are aware of their condition and diabetic group naturally exists. Cohort studies, which are not as reliable as RCTs, were therefore included in this meta-analysis. Secondly, the duration of diabetes for the diabetic patients, the surgical conditions, the surgeons, and the data collection techniques all work together to make some of the outcomes not uniform. Thirdly, most of the patients in the included studies are from Asia, which might be a potential source of deviations as corneal biomechanics may vary among races^57,58^.

## Method

### Search strategy

PubMed, EMBASE, and the Cochrane Controlled Trials Register were searched for relevant literature dated up to Jan 2017. The following terms were used to search for prospective studies in the selected databases: ((diabete OR diabetes) AND cataract surgery) AND corneal; ((‘cataract extraction’/exp OR ‘cataract extraction’) AND ‘diabetes mellitus’/exp OR ‘diabetes mellitus’) AND corneal; “diabetes” AND “cataract surgery” AND “corneal”. The bibliographies of the relevant review and original research articles were also scanned for potential trials that may have been missed in the primary searches.

### Study selection

The inclusion criteria for our selection process were set as follows: 1) Prospective controlled study, 2) the study included DM patients and normal patients who underwent phacoemulsification and IOL implantation, 3) the study reported at least one basic dataset of corneal properties, such as ECD, CV, HC%, and CCT, 4) the patients in the trials were absent of additional underlining diseases or eye disorders other than diabetes and cataract. Patients with other complications that could affect corneal state (e.g. severe liver or kidney dysfunction, glaucoma, iritis, or eye injury) were excluded.

### Screening process

Two reviewers, working independently from each other, first conducted preliminary reviews of the titles and abstracts; then, the full articles were analyzed to select the studies that met our predefined criteria. Disagreements between the two reviewers were resolved through careful discussion, involving a third or fourth reviewer when necessary, until a consensus was reached.

### Quality assessment

The Newcastle–Ottawa scale (NOS) was used for quality assessment. The NOS contains eight items (nine scores in total) which fit into three categories: selection (four scores), comparability (two scores), and exposure of a case-control study or outcome of a cohort study (three scores). A score ≥6 indicates good quality.

### Data extraction process

The patient data was extracted from the selected studies via a standard form: first author, country (province), year of publication, age of patient, sex of patient, follow up duration, quality control, and preoperative diabetes condition. The second reviewer double-checked all data.

The measurement of corneal properties included corneal endothelial cell density (ECD), corneal endothelial hexagon percentage (HC%), corneal endothelial coefficient of variation (CV), and central corneal thickness (CCT). Most of the studies only reported the absolute values of the outcomes at a preoperative baseline and postoperative time points. The mean and standard deviation outcomes of the corneal changes were calculated as follows:

1 $${\rm{\Delta }}mean=mea{n}_{post}-mea{n}_{pre}$$

2 $$S{D}_{{\rm{\Delta }}mean}=\sqrt{S{D}_{pre}^{2}+S{D}_{post}^{2}-2\rho S{D}_{pre}S{D}_{post}}$$

where *ρ* is the covariance coefficient, pre and post are short for preoperative and postoperative state. Generally, *ρ* was treated as ~0.5. The mean change percentages were calculated according to:

3 $${\rm{\Delta }}mean \% =\frac{{\rm{\Delta }}mean}{mea{n}_{pre}},\,S{D}_{{\rm{\Delta }}mean \% }=\frac{S{D}_{{\rm{\Delta }}mean}}{mea{n}_{pre}}$$

For situations when the selected study included multiple groups, a group-combining method^59^ was used in this meta-analysis to create a single pair-wise comparison. Considering a two-group combining process in which group 1 has a sample size of *N*_1_, a mean outcome of *M*_1_, and a standard derivation of SD_1_, and group 2 has similar (N_2,_ M_2_, and *SD*_2_), the combined group 1 + 2 would be calculated as:

4 $${N}_{1+2}={N}_{1}+{N}_{2};{M}_{1+2}=\frac{{N}_{1}{M}_{1}+{N}_{2}{M}_{2}}{{N}_{1}+{N}_{2}}$$

5 $$S{D}_{1+2}=\sqrt{\frac{({N}_{1}-1)S{D}_{1}^{2}+({N}_{2}-1)S{D}_{2}^{2}+\frac{{N}_{1}{N}_{2}}{{N}_{1}+{N}_{2}}{({M}_{1}-{M}_{2})}^{2}}{{N}_{1}+{N}_{2}-1}}$$

If there were more than two groups to combine, the strategy was to repeat this method sequentially (i.e. combine group 1 and group 2 to create group 1 + 2, and then combine group 1 + 2 and group 3 to create group 1 + 2 + 3, and so on).

### Statistical analysis

The statistical analysis was performed using Review Manager 5.3. The weighted mean difference (WMD) and 95% confidence interval (CI) were calculated from selected outcomes. P < 0.05 was considered statistically significant. Statistical heterogeneity was tested using the chi-squared and *I* ^2^ tests. A random-effect meta-regression model was used when significant heterogeneity (*I* ^2^ >  50%) or clinical divergence were found. Otherwise, a fixed-effect meta-regression model was chosen. Publication bias was measured in Begg’s and Egger’s test using Stata 14. To evaluate the stability and reliability of our pooled outcomes, a sensitivity analysis was performed using a one-study-removed analysis to assess whether the results were affected by the excessive weight of a single study.

## Electronic supplementary material

Supplementary Tables S1-S8
