# Supplementary material for: Clinical evaluation of corneal changes after phacoemulsification in diabetic and non-diabetic cataract patients, a systematic review and meta-analysis
Source: Sci Rep. 2017 Oct 26;7:14128. doi: 10.1038/s41598-017-14656-7 (PMC5658349; doi:10.1038/s41598-017-14656-7)
Supplement: Supplementary file 1 — Supplementary Tables S1-S8 [file 41598_2017_14656_MOESM1_ESM.docx]

**Title Page**

**Full title:**

**Clinical evaluation of corneal changes after phacoemulsification in diabetic and non-diabetic cataract patients, a systematic review and meta-analysis**

**Authors:** Yizhen Tang^1,2^, Xinyi Chen^1,2^, Xiaobo Zhang^1,2^, Qiaomei Tang^1,2^, Siyu liu^1,2^, Ke Yao^1,2^*

**Author information**

^1^Eye Center, Second Affiliated Hospital, School of Medicine, Zhejiang University, Hangzhou, Zhejiang, P.R.China

^2^Key Laboratory of Ophthalmology of Zhejiang Province, Hangzhou, P.R.China

*Correspondence: Ke YAO, MD, Professor and Chief. Eye Center, Second Affiliated Hospital, School of Medicine, Zhejiang University, No. 88 Jiefang Road, Hangzhou 310009, China. E-mail address: [xlren@zju.edu.cn](mailto:xlren@zju.edu.cn).

**Supplementary Information**

Supplementary Table S1 Comparison of phaco time in diabetic and non-diabetic patients.

Supplementary Table S2 Comparison of phaco energy in diabetic and non-diabetic patients

Supplementary Table S3 Comparison of visual acuity in diabetic and non-diabetic patients

Supplementary Table S4 Comparison of lens nucleus hardness in diabetic and non-diabetic patients

Supplementary Table S5. Pooled MD, heterogeneity and publication Bias.

Supplementary Table S6. Newcastle–Ottawa Scale for observational studies (cohorts).

Supplementary Table S7. phacoemulsification machines and examining machines in included studies.

Supplementary Table S8. Subgroup analysis of the examining machines.

**Table S1.** **Comparison of phaco time in diabetic and non-diabetic patients. DM = diabetes mellitus.**

| **Phaco time (s)** | **DM(SD, range)** | **Non-DM** | **P** |
| --- | --- | --- | --- |
| Wan 2015 | 22s | 20.5s | P > 0.05 |
| Zhu 2014 | 32.5s | 24.5s | P > 0.05 |
| Yan 2014 | 14–18min | |  |
| Su 2014 | 15–20s | |  |
| Liu 2014 | 26.8s | 22.5s | P > 0.05 |
| Zhan 2013 | 12s | |  |
| Yang 2011 | 1–3 min | |  |
| Wu 2010 | 12 min | |  |
| Morikubo 2004 |  | | P > 0.05 |

**Table S2.** **Comparison of phaco energy in diabetic and non-diabetic patients**

| **Phaco energy (%)** | **DM** | **Non-DM** | **P** |
| --- | --- | --- | --- |
| Wan 2015 | 12.3 | 9.5 | P > 0.05 |
| Zhu 2014 | 13.7 | 12.5 | P > 0.05 |
| Yan 2014 |  | | P > 0.05 |
| Su 2014 | 6–9% | |  |
| Liu 2014 | 21.3 | 18.1 | P > 0.05 |
| Zhao 2013 | 40% | |  |
| Yang 2011 | 19% | |  |
| Wu 2010 | 21% | |  |

**Table S3.** **Comparison of phaco energy in diabetic and non-diabetic patients**

| **BCVA** | **preoperative** |  | **postoperative** |  |
| --- | --- | --- | --- | --- |
|  | **DM** | **nDM** | **DM** | **nDM** |
| Misra 2015 | Log MAR 0.62(0.56) | Log MAR 0.53(0.42) | Log MAR 0.22(0.31) | Log MAR 0.12(0.14) |
| Zhu 2014 | P > 0.05 |  | P > 0.05 |  |
| Zhao 2013 | P > 0.05 |  | P > 0.05 |  |
| Wang 2013 |  |  | >0.3 50:<0.3 12 | >0.3 78:<0.3 4 |
| Yang 2011 | P > 0.05 |  | P > 0.05 |  |
| Hugod 2011 | P > 0.05 |  | P > 0.05 |  |
| Wu 2008 | P > 0.05 |  | P > 0.05 |  |

**Table S4.** **Comparison of lens nucleus hardness in diabetic and non-diabetic patients**

| **Lens nucleus hardness** | **DM（grade 2:3:4）** | **Non-DM** | **P** |
| --- | --- | --- | --- |
| Wan 2015 | 11:31:1 | 10:32:1 | P > 0.05 |
| Zhu 2014 | 2:50:4 | 4:52:4 | P > 0.05 |
| Yan 2014 | Grade 3–4 | |  |
| Liu 2014 | Grade 2–3 | |  |
| Su 2014 | 0:52:0 | 0:33:0 |  |
| Yang 2011 |  | | P > 0.05 |

**Table S5. Pooled MD, Heterogeneity and Publication Bias in Meta-analysis: Comparison between diabetic and non-diabetic patients.**

| **Outcome** | **No. of trials** | **WMD (95% CI)** | **P value** | **Heterogeneity** | | **Publication bias** | |
| --- | --- | --- | --- | --- | --- | --- | --- |
|  |  |  |  | **I^2^** | **P_heterogeneity_** | **Begg** | **Egger** |
| **Central Corneal thickness** |  |  |  |  |  |  |  |
| preoperative | 4 | 2.86 [-3.65, 9.37] | 0.39 | 37% | 0.19 | 0.089 | 0.057 |
| postoperative 1 week | 3 | 17.96 [5.24, 30.68] | 0.006 | 82% | 0.004 | 1 | 0.617 |
| postoperative 1 month | 3 | 22.59 [10.23, 34.94] | <0.001 | 78% | 0.01 | 1 | 0.973 |
| postoperative 3 months | 3 | 12.92 [9.22, 16.63] | <0.001 | 0% | 0.4 | 1 | 0.211 |
| **Increase in corneal thickness%** |  |  |  |  |  |  |  |
| postoperative 1 week | 3 | 2.81 [-0.36, 5.98] | 0.08 | 98% | <0.001 | 1 | 0.446 |
| postoperative 1 month | 3 | 3.86 [1.28, 6.45] | 0.003 | 97% | <0.001 | 1 | 0.325 |
| postoperative 3 months | 3 | 1.56 [-0.57, 3.70] | 0.15 | 89% | <0.001 | 1 | 0.955 |
| **Endothelial cell density** |  |  |  |  |  |  |  |
| preoperative | 13 | -98.60 [-181.39, -15.82] | 0.02 | 93% | <0.001 | 0.855 | 0.301 |
| postoperative 1 day | 6 | -129.29 [-149.47, -109.10] | <0.001 | 0% | 0.51 | 0.133 | 0.301 |
| postoperative 1 week | 10 | -192.17 [-267.04, -117.29] | <0.001 | 91% | <0.001 | 0.283 | 0.448 |
| postoperative 1 month | 10 | -205.53 [-258.30, -152.76] | <0.001 | 76% | <0.001 | 0.592 | 0.429 |
| postoperative 3 months | 9 | -229.83 [-283.54, -176.12] | <0.001 | 76% | <0.001 | 0.602 | 0.211 |
| **Endothelial cell loss%** |  |  |  |  |  |  |  |
| postoperative 1 day | 6 | 3.40 [1.82, 4.97] | <0.001 | 86% | <0.001 | 1 | 0.264 |
| postoperative 1 week | 10 | 3.45 [2.64, 4.25] | <0.001 | 39% | 0.1 | 0.721 | 0.122 |
| postoperative 1 month | 10 | 3.80 [1.84, 5.75] | <0.001 | 89% | <0.001 | 0.858 | 0.777 |
| postoperative 3 months | 9 | 4.85 [1.60, 8.10] | 0.003 | 93% | <0.001 | 0.917 | 0.811 |
| **Coefficient of variation** |  |  |  |  |  |  |  |
| preoperative | 8 | 2.62 [1.41, 3.83] | <0.001 | 77% | <0.001 | 0.063 | 0.240 |
| postoperative 1 day | 3 | 4.75 [-1.51, 11.00] | 0.14 | 98% | <0.001 | 1 | 0.860 |
| postoperative 1 week | 6 | 5.09 [2.68, 7.51] | <0.001 | 89% | <0.001 | 0.260 | 0.584 |
| postoperative 1 month | 7 | 6.71 [3.60, 9.81] | <0.001 | 97% | <0.001 | 0.548 | 0.339 |
| postoperative 3 months | 6 | 6.65 [3.14, 10.15] | <0.001 | 96% | <0.001 | 1 | 0.088 |
| **Coefficient of variation changes** |  |  |  |  |  |  |  |
| postoperative 1 day | 3 | 3.27 [-3.02, 9.56] | 0.31 | 98% | <0.001 | 1 | 0.908 |
| postoperative 1 week | 6 | 2.02 [0.66, 3.39] | 0.004 | 64% | 0.02 | 0.707 | 0.625 |
| postoperative 1 month | 7 | 3.68 [0.47, 6.88] | 0.02 | 97% | <0.001 | 1 | 0.129 |
| postoperative 3 months | 6 | 3.24 [-1.06, 7.53] | 0.14 | 98% | <0.001 | 0.707 | 0.080 |
| **Hexagonal cells%** |  |  |  |  |  |  |  |
| preoperative | 11 | -3.68 [-5.52, -1.84] | <0.001 | 84% | <0.001 | 0.755 | 0.880 |
| postoperative 1 day | 5 | -6.52 [-8.01, -5.03] | <0.001 | 62% | 0.03 | 0.462 | 0.567 |
| postoperative 1 week | 9 | -8.10 [-10.76, -5.44] | <0.001 | 87% | <0.001 | 0.754 | 0.621 |
| postoperative 1 month | 9 | -8.55 [-11.56, -5.53] | <0.001 | 92% | <0.001 | 0.917 | 0.664 |
| postoperative 3 months | 7 | -8.90 [-11.84, -5.97] | <0.001 | 92% | <0.001 | 0.764 | 0.221 |
| **Hexagonal cells% loss** |  |  |  |  |  |  |  |
| postoperative 1 day | 5 | 3.70 [2.25, 5.16] | <0.001 | 54% | 0.07 | 0.462 | 0.478 |
| postoperative 1 week | 9 | 3.98 [2.49, 5.48] | <0.001 | 56% | 0.02 | 0.466 | 0.279 |
| postoperative 1 month | 9 | 4.31 [2.23, 6.40] | <0.001 | 84% | <0.001 | 0.466 | 0.340 |
| postoperative 3 months | 7 | 4.56 [2.32, 6.80] | <0.001 | 86% | <0.001 | 0.368 | 0.203 |

**Table S6. Newcastle–Ottawa Scale for observational studies.**

| **Study ID** | **Study design** | **Selection** | **Comparability** | **Outcome** | **Total** |
| --- | --- | --- | --- | --- | --- |
| Li 2016 | controlled study | *** |  |  | 8 |
| Misra 2015 | cohort study | *** |  |  | 8 |
| Zhu 2014 | controlled study | *** |  |  | 8 |
| Yan 2014 | controlled study | *** |  |  | 8 |
| Su 2014 | controlled study | *** |  |  | 8 |
| Liu 2014 | controlled study | *** |  |  | 8 |
| Zhao 2013 | controlled study | *** |  |  | 8 |
| Wang 2013 | controlled study | *** |  |  | 7 |
| Yang 2011 | controlled study | *** |  |  | 7 |
| Hugod 2011 | controlled study | *** |  |  | 8 |
| Wu 2010 | controlled study | *** |  |  | 8 |
| Wu 2008 | controlled study | *** |  |  | 8 |
| Morikubo 2004 | controlled study | *** |  |  | 8 |

A higher overall score corresponds to a lower risk of bias; a score of six or more (out of nine) indicates a low risk of bias. Each * equals 1 points.

**Table S7. phacoemulsification machines and examining machines in included studies.**

| **Studies** | **phacoemulsification machines** | **Cornea examining machines** |
| --- | --- | --- |
| Li 2016 | Alcon Infiniti Phaco System | Confoscan 4 (NIDEK Co, Ltd, Aichi, Japan) |
| Misra 2015 | Bausch & Lomb, Rochester, NY | Confoscan 4, NIDEK |
| Zhu 2014 | [AMOSovereign Compact Phacoemulsification System](https://www.baidu.com/link?url=Y9o8J8zsgR5DmlHtf-mutwyoAlNKxMLvJzFBtL8ov3zFgphdMW_l0Y3RgAujKr1Ns3BdD1-oelt8Vjiftf8JdfZo_DPPibyb52XWuq88COe&wd=&eqid=f4a3cb950000647c0000000659900a29) | noncontact specular microscope (SP 3000P; Topcon, Tokyo, Japan) |
| Yan 2014 | Bausch & Lomb, Rochester, NY | SP 3000P; Topcon |
| Su 2014 | Alcon Infiniti Phaco System | SP 2000P; Topcon |
| Liu 2014 | Alcon Infiniti Phaco System | SP 2000P; Topcon |
| Zhao 2013 | Alcon Infiniti Phaco System | Confoscan 4, NIDEK |
| Wang 2013 | Bausch & Lomb, Rochester, NY | SP 2000P; Topcon |
| Yang 2011 | Alcon Infiniti Phaco System | SP 2000P; Topcon |
| Hugod 2011 | Storz Millennium phaco machine | SP 2000P; Topcon |
| Wu 2010 | Alcon Infiniti Phaco System | SP 2000P; Topcon |
| Wu 2008 | Alcon Infiniti Phaco System | EM-100, Tomey, Aichi, Japan |
| Morikubo 2004 | Alcon Infiniti Phaco System | Noncontact specular microscope,NONCONROBO(Konan Medical, Inc, Hyogo, Japan). T |

|  | **Group 1** | | **Group2** | | **Test for subgroup differences** | |
| --- | --- | --- | --- | --- | --- | --- |
|  | **MD** | **I^2^** | **MD** | **I^2^** | **P** | **I^2^** |
| ECD pre | -74.33 [-135.66, -12.99] | 40% | -133.57 [-272.50, 5.37] | 96% | 0.44 | 0% |
| ECD post 1d | -157.41 [-195.55, -119.28] | 0% | -118.34 [-142.13, -94.54] | 0% | 0.09 | 65.6% |
| ECD post 1w | -196.76 [-228.95, -164.56] | 0% | -201.58 [-323.71, -79.45] | 94% | 0.94 | 0% |
| ECD post 1m | -280.64 [-351.73, -209.55] | 34% | -166.88 [-211.17, -122.58] | 53% | 0.008 | 85.9% |
| ECD post 3m | -262.45 [-362.53, -162.37] | 53% | -204.90 [-255.05, -154.75] | 67% | 0.31 | 1.5% |
| ECD loss post 1d | 3.26 [-0.91, 7.42] | 78% | 3.97 [2.66, 5.28] | 82% | 0.75 | 0% |
| ECD loss post 1w | 2.94 [1.52, 4.36] | 0% | 3.49 [2.50, 4.48] | 54% | 0.53 | 0% |
| ECD loss post 1m | 7.11 [3.72, 10.50] | 64% | 2.12 [-0.29, 4.53] | 92% | 0.02 | 81.9% |
| ECD loss post 3m | 6.59 [4.86, 8.32] | 7% | 3.02 [-2.29, 8.32] | 96% | 0.21 | 36.5% |
| HC pre | -3.65 [-6.61, -0.68] | 72% | -3.64 [-6.39, -0.90] | 88% | 1.00 | 0% |
| HC post 1d | -6.04 [-6.84, -5.23] | 0% | -6.95 [-10.48, -3.42] | 79% | 0.62 | 0% |
| HC post 1w | -8.67 [-15.39, -1.95] | 78% | -7.81 [-10.87, -4.76] | 90% | 0.82 | 0% |
| HC post 1m | -9.02 [-14.31, -3.73] | 84% | -8.33 [-12.33, -4.32] | 95% | 0.84 | 0% |
| HC post 3m | -6.30 [-10.56, -2.04] | 87% | -10.81 [-14.49, -7.13] | 90% | 0.12 | 59.5% |
| HC loss post 1d | 3.25 [2.26, 4.23] | 0% | 4.64 [1.19, 8.09] | 77% | 0.45 | 0% |
| HC loss post 1w | 4.34 [1.40, 7.29] | 0% | 3.99 [2.14, 5.84] | 71% | 0.84 | 0% |
| HC loss post 1m | 3.21 [1.63, 4.79] | 0% | 4.71 [1.75, 7.67] | 90% | 0.38 | 0% |
| HC loss post 3m | 1.99 [0.87, 3.12] | 0% | 6.47 [2.81, 10.12] | 91% | 0.02 | 81% |
| CV pre | 4.60 [-0.05, 9.25] | 93% | 1.84 [1.07, 2.62] | 0% | 0.25 | 23.8% |
| CV post 1d | / | / | / | / | / | / |
| CV post 1w | / | / | / | / | / | / |
| CV post 1m | 8.56 [5.96, 11.16] | 71% | 6.09 [1.73, 10.45] | 97% | 0.34 | 0% |
| CV post 3m | 7.26 [1.41, 13.12] | 96% | 5.99 [1.79, 10.19] | 92% | 0.73 | 0% |
| CV change post 1d | / | / | / | / | / | / |
| CV change post 1w | / | / | / | / | / | / |
| CV change post 1m | 2.36 [-8.58, 13.29] | 98% | 4.10 [0.63, 7.58] | 94% | 0.77 | 0% |
| CV change post 3m | 2.48 [-5.71, 10.68] | 99% | 3.92 [0.05, 7.79] | 91% | 0.76 | 0% |

**Table S8. Subgroup analysis of the examining machines.**

**(Group1 contains studies using SP2000p Topcon. The rest are group2. ECD: endothelial cell density, HC: hexagonal cell, CV: coefficient of variance. pre: preoperative, post: postoperative, MD: mean difference, I2 = extent of inconsistency, /: not enough studies)**
